# Auditory steady state response can predict declining EF in healthy elderly individuals

**DOI:** 10.3389/fnagi.2025.1516932

**Published:** 2025-02-04

**Authors:** Xiaopeng Mao, Nelly Shenton, Sadasivan Puthusserypady, Martin Johannes Lauritzen, Krisztina Benedek

**Affiliations:** ^1^Department of Health Technology, Technical University of Denmark, Kongens Lyngby, Denmark; ^2^Department of Clinical Neurophysiology, Zealand University Hospital, Roskilde, Denmark; ^3^Faculty of Health and Medical Sciences, University of Copenhagen, Copenhagen, Denmark

**Keywords:** electroencephalogram (EEG), auditory steady state response (ASSR), gamma-band, cognitive decline, executive function (EF), machine learning (ML)

## Abstract

**Background:**

The aging population imposes significant economic and societal challenges, underscoring the need for early detection of individuals at risk of cognitive decline prior to the onset of clinical symptoms. This study explores the association between gamma-band Auditory Steady-State Responses (ASSRs) and subclinical cognitive decline using longitudinal data from healthy volunteers in the Metropolit Birth Cohort (MBC).

**Methods:**

Longitudinal recordings of cognitive test results and ASSRs at 40 Hz stimulation were analyzed. Generalized Linear Models (GLMs) were employed to determine the association between ASSR characteristics and cognitive performance with an emphasis on Executive Function (EF) at ages 61–68. Additionally, Vision Transformers (ViTs) were trained to distinguish between individuals with declining and stable cognitive performance.

**Results:**

Subjects with declining cognitive performance through midlife showed a larger area of entrainment and delayed neural assembly of ASSRs compared to those with stable cognitive performance. These neurophysiological changes were correlated with poorer EF, as measured by the Stockings of Cambridge (SOC) task. The ViTs trained and cross-validated on time-frequency-transformed Electroencephalograms (EEGs) achieved an average cross-subject accuracy of 51.8% in identifying cognitive decline.

**Conclusion:**

Gamma-band ASSR characteristics are linked to early cognitive decline in middle-aged individuals, offering potential as biomarkers. However, the limited predictive accuracy of ML models emphasizes the need for further refinement to enhance their clinical applicability.

## 1 Introduction

Healthy aging is a major achievement for society, but it comes with significant challenges in delivering healthcare and supporting the wellbeing of a growing elderly population. It is crucial to ensure that these extra years of life are not only free from serious illnesses but also marked by good mental and physical health. This will help reduce the heavy economic and social pressures of an aging population and contribute to a healthier, more sustainable future for everyone l (Livingston et al., 2020). Longitudinal and multimodal biomarker studies have demonstrated that Alzheimer's Disease (AD) encompasses a prolonged latent phase known as preclinical AD, which occurs decades before the onset of symptoms. Treating AD during this preclinical phase presents an ideal opportunity to slow down the disease progression. However, designing clinical trials for this population remains a complex challenge (Rafii and Aisen, 2023). Detecting cognitive decline years before memory loss begins could allow for early interventions, potentially changing the course of this challenging condition. However, diagnosing cognitive decline early is difficult due to issues like low reliability, invasive methods, and high costs.

Research shows a possible connection between hearing loss and dementia, with estimates suggesting that hearing loss might contribute to about 9% of dementia cases (Livingston et al., 2020). Based on our earlier findings (Wiegand et al., 2018; Horwitz et al., 2019, 2017), our current study aims to identify connections between preclinical cognitive decline and Auditory Steady-State Responses (ASSRs). To address these challenges, this study seeks to find the correlation between ASSRs and EF. EF is a complex cognitive control responsible for making adaptive changes in physical and social environments. It consists of sub-components, such as inhibition, shifting, and updating working memory (Miyake et al., 2000). A prominent feature of cognitive aging is the decline of EF abilities. Numerous studies have reported that older adults perform poorer than the younger in such tasks (Idowu and Szameitat, 2023; Hasher and Zacks, 1988). The main question is how and why do brain networks deteriorate differently during the lifespan and what controls the differences between high-functioning and declining individuals? The ASSR is a result of entrainment of the brain's oscillatory activity to the frequency and phase of temporally modulated stimuli.

In this study, we hypothesize that ASSRs can distinguish and predict subjects with declining cognition. Our objective is to identify changes in perceptive networks that predict cognitive decline. Building on these insights, we have also trained a state-of-the-art Machine Learning (ML) algorithm to detect healthy middle-aged individuals at risk of cognitive deterioration. By leveraging Deep Learning (DL) techniques with accessible Electroencephalography (EEG) technology, we investigate the possibility of a cost-effective solution. Furthermore, interpreting DL models can reveal deep insights into the underlying mechanisms of the disease, enhancing our understanding of its progression and facilitating early detection (Kim et al., 2023; Sibilano et al., 2023).

## 2 Subjects and methods

**Figure 1:**
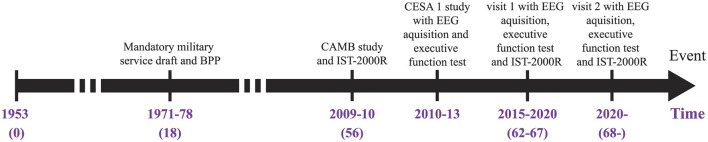
Timeline of the studies behind the provided data. The intelligence tests are from BPP and IST-2000R while the two CESA studies investigate the ASSRs and EF. BPP, Børge Priens Prøve; IST-2000R, Intelligens Struktur Test 2000 Revised; CAMB, Copenhagen Aging and Midlife Biobank.

Participants for this study were selected from the Metropolit Danish male Birth Cohort (MBC), which includes 11,532 men born in 1953 in the Copenhagen Municipality region (Osler et al., 2006). The cohort was cognitively assessed at the age of 18 years as part of the Danish draft board examination using Børge Priens Prøve (BPP), an Intelligence Quotient (IQ) test consisting of 4 paper-pencil subtests involving logical, verbal, numerical, and spatial reasoning (Teasdale, 2009; Teasdale et al., 2011). A subset of participants of the original sample was assessed again at the age of 56 years as part of the Copenhagen Aging and Midlife Biobank (CAMB) project (Avlund et al., 2014; Lund et al., 2016; Mortensen et al., 2014). In the CAMB project, IQ was measured using a version of the Intelligens Struktur Test 2000 Revised (IST-2000-R), which included 3 subtests involving verbal analogies, number series, and sentence completion. Linear regression was generated between cognitive scores at youth (BPP+IQ) (18 years) and (IST-2000-R total test score) in late-middle age (56 years) (Osler et al., 2006; Wiegand et al., 2018). Participants (*n* = 178) for the present study were selected among those with stable cognitive function (*n* = 83) and the cognitively declining group (*n* = 95) using a BPP and an IST-2000-R test. We retrospectively collected ASSR recordings between 2014 and 2016 as part of the CESA 2 study. Figure 1 illustrates an overview of the data acquisition process.

### 2.1 Standard protocol approvals, registrations, and patient consents

The study was approved by the Capital Region of Denmark's Health Research Ethics Committee (H-1–2014032) and conducted according to the Declaration of Helsinki. All participants provided written informed consent regarding their participation and publication of the current data.

### 2.2 Recordings

EEG was recorded with a 64-channel elastic Quick-Cap connected to a Neuroscan bio-amplifier (SynAmpsRT, Compumedics, http://compumedicsneuroscan.com/). Electrodes were placed according to the international 10-20 system. Curry7 (http://compumedicsneuroscan.com/) (Curry version 7.0.12) was used to record EEG signals with a sampling frequency of 2 kHz. All EEG electrodes were referenced to a physical reference between Cz and Cpz. The ground electrode was between Fz and Fpz. Two horizontal Electrooculography (EOG) electrodes were positioned laterally to the right and left eyes, while two vertical EOG electrodes were placed above and below the left eye. The Electrocardiogram (ECG) and Electromyography (EMG) electrodes were included to detect and remove ECG and muscle artifacts from the EEG signal during signal processing. For the ECG, one electrode was placed just under the right clavicle and the other at the left lower chest. In addition, two electrodes were placed under the chin lateral to the midline for the EMG.

Participants were exposed to auditory stimuli delivered through noise-isolating headsets and controlled by a separate computer using the STIM2 program (developed by Compumedics Neuroscan for precise stimulus presentation). The experiment took place in a shielded medical examination room at Rigshospitalet, Glostrup, Denmark. During the session, participants were seated in front of a monitor, focusing on a red fixation cross while listening to a sequence of clicks. These clicks used a 1 kHz carrier frequency and were amplitude-modulated at 40 Hz. For each participant, 40 trials were conducted, with each trial consisting of 6 seconds of auditory stimulation recorded continuously using EEG. The interval between trials (Inter-Trial Interval or ITI) was set at 5 seconds, resulting in a total session duration of approximately 7 minutes and 15 seconds per participant.

### 2.3 Signal analysis

Preprocessing and artifact reduction were carried out using the EEGLAB v.2023.1 (Delorme and Makeig, 2004; Nagabhushan Kalburgi et al., 2024) toolbox in MATLAB (R2022a, MathWorks, Natick, MA, USA). All EEG electrodes were re-referenced to the common average and downsampled to 250 Hz. A Chebyshev type 2 Infinite Impulse Response (IIR) band-pass filter of order 18 was applied to filter the EEG between 0.5 and 90 Hz. A Chebyshev type 2 IIR notch-filter of order 8 was used to filter the 50 Hz power-line interference. Both filters were applied with zero phase using the MATLAB function filtfilt.

**Figure 2:**
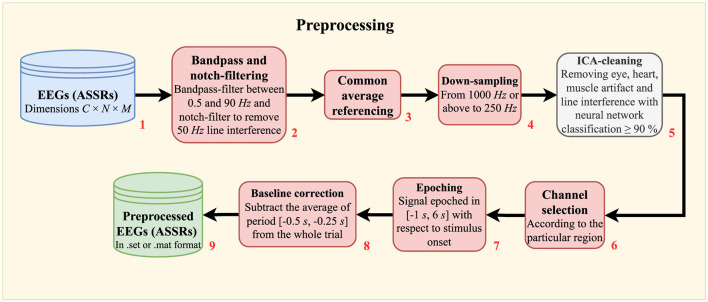
Flow diagram showing the raw EEG preprocessing. In short, the procedures involve noise minimization (filtering in block 2, average referencing in block 3, and ICA-cleaning in block 5), down-sampling (block 4), epoching (block 7), and baseline correction (block 8). *C*: number of EEG channels. *N*: number of data points. *M*: number of epochs.

Independent Component Analysis (ICA) was applied to detect and remove components that contain eye blinks and muscle artifacts with ≥90% classification accuracy, respectively, using the ICLabel plugin in EEGLAB, which automatically classified the source of the independent components. A mixed brain region was selected and included the channels FT7, T7, TP7, P7, P5, Fz, FCz, Cz, CPz, FT8, T8, TP8, P6, and P8. This selection reflects the physiological behavior of the brain toward the auditory stimulus (Purves et al., 2019; Parciauskaite et al., 2019). The frontal region with the channels F7, F5, F3, F1, Fz, F2, F4, F6, and F8 was also investigated due to the implication of the frontal region in EF (Stuss, 2011). Additionally, we investigated the whole head to look into the ASSR power distribution and fluctuations. The EEG data was epoched around the stimulus period (–1 s to 6 s relative to stimulus onset) and then baseline-corrected (–0.5 s to –0.25 s relative to stimulus onset). By denoting the stimulus onset time with *t*_0_, the event with *a* and the EEG channel with *i*, each epoch is defined as:


(1) $$\begin{array}{l} epoch_{EEG}=\left[x_{i,t_{0}-1s}^{\left(a\right)},x_{i,t_{0}+6s}^{\left(a\right)}\right]-baseline, \end{array}$$

where *x*_*i*_ is a single-channel EEG from the EEG matrix **E**. *baseline* represents the average background EEG activity and is used to correct the baseline shift of the ASSR (Kashiwase et al., 2012; Parciauskaite et al., 2019; Nam et al., 2018):


(2) $$\begin{array}{l} baseline=𝔼\left\{\left[x_{i,t_{0}-0.5s}^{\left(a\right)},x_{i,t_{0}-0.25s}^{\left(a\right)}\right]\right\}, \end{array}$$

where 𝔼{·} is the expectation operator. The flow diagram in Figure 2 details all the signal processing steps.

#### 2.3.1 Dimensionality reduction using Rhythmic Entrainment Source Separation (RESS)

The preprocessed EEG has 14 channels for the mixed region and 9 channels for the frontal region. To enhance the Signal-to-Noise-Ratio (SNR) and to use the information in all available channels, a spatial filtering technique entitled Rhythmic Entrainment Source Separation (RESS) was applied (Cohen and Gulbinaite, 2017). In brief, RESS uses the covariance matrix of the peak stimulus frequency (40 Hz), denoted S and the covariance matrix of the neighboring frequencies, denoted R, in an eigen-decomposition to enhance the SNR. More specifically, the eigen-decomposition is applied to R^−1^S to calculate the matrix V, which contains the spatial filters as eigenvectors (Cohen and Gulbinaite, 2017):


(3) $$\begin{array}{l} {\text{R}}^{-1}\text{S}=\text{VΛ}{\text{V}}^{-1}, \end{array}$$

where Λ is a diagonal matrix containing the corresponding eigenvalues. In practice, however, V is found by solving SV = RVΛ for numerical stability:


(4) $$\begin{array}{l} \text{SV}=\text{RVΛ }\Leftrightarrow \;{\text{R}}^{-1}\text{SV}=\text{VΛ }\Leftrightarrow \;{\text{R}}^{-1}\text{S}=\text{VΛ}{\text{V}}^{-1}. \end{array}$$

It is important to notice that R^−1^S is non-symmetric, so the eigenvectors are non-orthogonal compared to e.g. the eigenvectors from PCA (Cohen and Gulbinaite, 2017). The signal length for ASSR is selected as the total duration of the stimulation i.e., 6 s because this gives the greatest SNR calculated from non-stimulation frequencies. The Full Width at Half Maximum (FWHM) of 40 Hz is set to 0.5 Hz. The distance of neighboring frequencies is set to 1 Hz, and the FWHM of the neighboring frequencies is set to 1 Hz. The eigenvector corresponding to the largest eigenvalue is transposed and multiplied with the EEG matrix. As a result, a single-channel time series is returned for each trial with accentuated 40 Hz content, which can be processed using the so-called Complex Demodulation (CD).

#### 2.3.2 Calculation of average ASSR power

We used the MATLAB function bandpower to compute the power for the 40 Hz signal in each epoch. Subsequently, we calculated the average power over all signal epochs. The ASSR power was calculated as the average power estimate for the mixed region at the stimulation frequency of 40 Hz.

#### 2.3.3 Complex demodulation of the signal

CD is a fundamental signal processing technique used to extract the temporal characteristics of a signal (Puthusserypady, 2021; Richard et al., 2020; Kashiwase et al., 2012; Draganova and Popivanov, 1999).

The temporal characteristics include an envelope *A*(*t*) and a phase ϕ(*t*) of the real and continuous RESS signal *R*(*t*):


(5) $$\begin{array}{l} R\left(t\right)=A\left(t\right)\cos\left[2\pi ft+\phi \left(t\right)\right]+N\left(t\right)\\ =A\left(t\right)\frac{e^{j\left[2\pi ft+\phi \left(t\right)\right]}+e^{-j\left[2\pi ft+\phi \left(t\right)\right]}}{2}+N\left(t\right), \end{array}$$

where *f* is the frequency of the signal, *t* is the continuous time, and *N*(*t*) is the noise from all frequencies except the 40 Hz (Kashiwase et al., 2012). Sometimes, *N*(*t*) strongly reduced the quality of the CD. Hence, a narrow-band Chebyshev type 2 IIR bandpass filter was used to remove *N*(*t*) before the next steps:


(6) $$\begin{array}{l} R_{BP}\left(t\right)=R\left(t\right)⊗h_{BP}\left(t\right)\approx A\left(t\right)\frac{e^{j\left[2\pi ft+\phi \left(t\right)\right]}+e^{-j\left[2\pi ft+\phi \left(t\right)\right]}}{2}, \end{array}$$

where the “⊗” sign denotes convolution, *h*_*BP*_(*t*) is the impulse response function of the selected bandpass filter with cutoff frequencies of 39.5 and 40.5 Hz. The sharp transition bands of a Chebyshev type 2 IIR filter are beneficial to preserving the 40 Hz signal without distorting it too much.

To extract the amplitude and phase modulations from the bandpass-filtered *R*_*BP*_(*t*), the signal is frequency-shifted by multiplying a linear combination of sine and cosine functions (Puthusserypady, 2021; Kashiwase et al., 2012; Draganova and Popivanov, 1999):


(7) $$\begin{array}{l} {\tilde{R}}_{BP}\left(t\right)=R_{BP}\left(t\right)e^{-j2\pi ft}=A\left(t\right)\frac{e^{j\phi \left(t\right)}}{2}+A\left(t\right)\frac{e^{-j\left[4\pi ft+\phi \left(t\right)\right]}}{2}, \end{array}$$

where ${\tilde{R}}_{BP}\left(t\right)$ is a complex analytic signal (Puthusserypady, 2021). We then applied a lowpass filter to reduce the remaining noise in Equation 7:


(8) $$\begin{array}{l} {\tilde{R}}_{\text{filt}}\left(t\right)={\tilde{R}}_{BP}\left(t\right)⊗h_{LP}\left(t\right)=A\left(t\right)\frac{e^{j\phi \left(t\right)}}{2}, \end{array}$$

where *h*_*LP*_(*t*) denotes the impulse response function of the lowpass filter. We used a Chebyshev type 2 IIR lowpass filter with a cutoff frequency of 2 Hz. This is because its sharp transition bands were advantageous in producing smooth modulation profiles with appropriate amplitudes. ${\tilde{R}}_{\text{filt}}$ is therefore the filtered and processed RESS EEG signal.

The final step is to calculate the Amplitude Modulation (AM) and phase modulation (ITPC), which together constitute the CD (Kashiwase et al., 2012):


(9) $$\begin{array}{l} AM\left(t\right)=\left[\frac{1}{K}\overset{K}{\underset{k=1}{\sum }}2|{\tilde{R}}_{\text{filt}}\left(t,k\right)|\right]-baseline_{\text{AM}}, \end{array}$$

(10) $$\begin{array}{l} ITPC\left(t\right)=|\frac{1}{K}\overset{K}{\underset{k=1}{\sum }}\frac{{\tilde{R}}_{\text{filt}}\left(t,k\right)}{\left|{\tilde{R}}_{\text{filt}}\left(t,k\right)\right|}|-baseline_{\text{ITPC}}, \end{array}$$

where *k* denotes one single epoch in the total *K* epochs of ${\tilde{R}}_{\text{filt}}$. Physiologically, amplitude and phase modulation reveal different aspects of the neural response toward a stimulus. AM describes the magnitude and speed of the Action Potential (AP) generated by the neurons. Hence, the magnitude of AM increases when the neurons depolarize simultaneously, and it decreases if the neurons depolarize asynchronously (Richard et al., 2020). ITPC, on the other hand, describes the consistency of the neural synchronization across the EEG trials and varies between 0 and 1 (Kashiwase et al., 2012).

#### 2.3.4 Modified Cumulative Gaussian function

**Figure 3:**
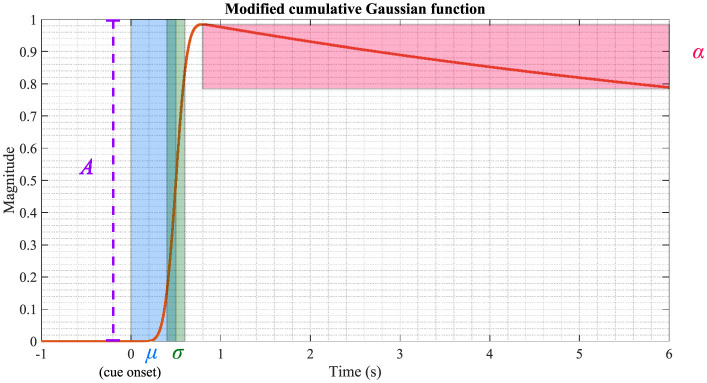
Visualization of the MCGF. The parameters *A*, μ, σ, and α are highlighted using different colors. The parameters used in this figure are: *A* = 1, *d* = 1, μ = 0.5, σ = 0.1 and α = 0.1.

In other related studies, a Modified Cumulative Gaussian function (MCGF) has been fitted to both the amplitude and phase modulation (Richard et al., 2020; Kashiwase et al., 2012). It is a linear combination of two cumulative Gaussian functions with five parameters:


(11) $$\begin{array}{l} MCGF\left(t\right)=\frac{Ad}{d+1}e^{\left\{\mu \alpha +0.5\sigma^{2}\alpha^{2}-\alpha t\right\}}G\left(t,\mu +\sigma^{2}\alpha ,\sigma \right)\\ +\frac{A}{d+1}G\left(t,\mu ,\sigma \right), \end{array}$$

where *A* is the amplitude, *d* is the ratio between the first and second term of the function, and *G*(·) is the normal cumulative Gaussian function with mean μ and standard deviation σ. α is the inverse time constant and decay of the function. Please note that *MCGF*(*t*) is used to estimate and visually display these parameters after *MCGF*(*t*) is fitted to AM or ITPC. Physiologically, *A* denotes the magnitude of AM or ITPC. μ denotes the latency, and σ is the slope of the fitted curve. α is used to investigate attentional behavior after the stimulus onset. Figure 3 illustrates an MCGF with the parameters highlighted in their respective colors. In practice, the period before the stimulus onset is set to zero, and the MCGF is fitted between 0 s and 5 s. In this study, all these five parameters are applied to model AM and ITPC. However, the main focus is on the parameters *A*, μ, and their interaction. The next step is investigating the correlations between the ASSR parameters, cognition, and EF.

### 2.4 Stockings Of Cambridge

The Stockings of Cambridge (SOC) test is a key component of the CANTAB test battery developed by Cambridge Cognition, used to evaluate EF, particularly strategic thinking and planning. In this study, the SOC test was the primary focus. Participants were shown two displays, each with three colored balls and three positions, known as “stockings,” where the balls can be placed. The test had two phases: in the “copy” phase, participants replicated a pattern from the upper display to the lower one, and in the “follow” phase, they mimicked the previous movements (Robbins et al., 1998; Coull et al., 1995). The test measuring variables like initial thinking time, subsequent thinking time, number of moves, and problems solved in minimum moves, etc., all reflect the participant's cognitive processing efficiency.

**Figure 4:**
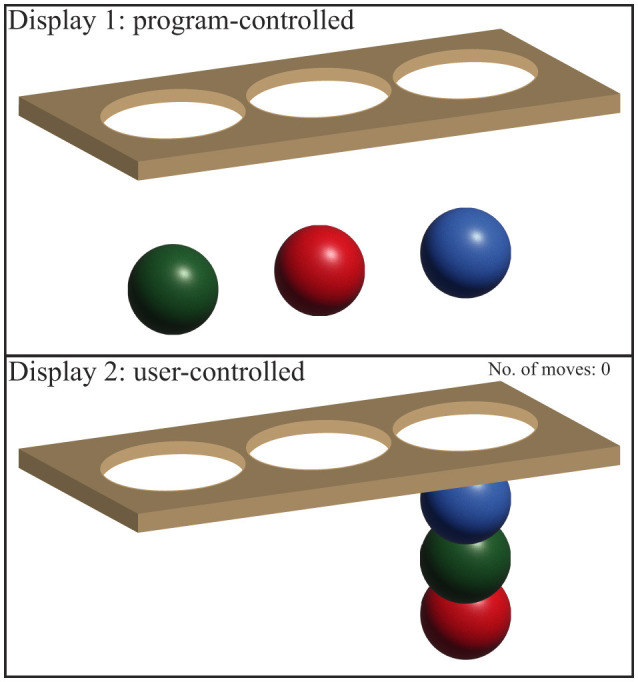
Displays for the SOC test. Display 1 shows a particular pattern, which the subject needs to copy in Display 2 by moving the balls into the stockings. The image is inspired by Cambridge Cognition web page for SOC.

EF is investigated using the SOC test (as part of the CANTAB test battery provided by Cambridge Cognition). During this test, two displays are shown to the subject (see Figure 4). Both displays contain three balls of different colors and three spaces (called stockings), which the balls can be put into. The upper display contains a particular pattern, which the subject needs to copy on the lower display by moving around the balls. This is called the “*copy*” phase, which involves strategic thinking and planning. Subsequently, the upper display would move the balls the same way as the subject has just done while the subject now needs to follow suit and move the balls in the same way in the lower display. This is called the “*follow*” phase. The “follow” phase is intended to record the time taken to initiate the movement and the time of the actual execution (Robbins et al., 1998; Coull et al., 1995). As for the outcome measures, the following variables are recorded:

- **Mean initial thinking time for *n*-move problems:** The mean initial thinking time refers to the mean time taken before making the first move in a *n*-move problem. The time of the “follow” phase is subtracted from the time of the “copy” phase. If the time of the “follow” phase is longer than the time of the “copy” phase, the mean initial thinking time becomes zero.
- **Mean subsequent thinking time for *n*-move problems:** The mean time after the first ball is selected until an *n*-move problem is completed divided by the total number of moves made. Likewise, this variable becomes zero if the time of the “follow” phase is longer than the time of the “copy” phase.
- **Mean moves for *n*-move problems:** The mean number of moves the subject makes to complete a problem requiring minimum *n* moves. The lower the outcome is, the better the performance is.
- **Problems solved in minimum moves:** The number of problems the subject has solved in minimum moves. A maximum of 12 problems can be achieved for this outcome measure. Hence, the more problems are solved, the better the performance is.

For the above-mentioned SOC variables, the number *n* is limited to *n* ∈ [2, 3, 4, 5]. The SOC test was taken for all participants during the same visit when their ASSR was recorded.

#### 2.4.1 Statistical tests

**Figure 5:**
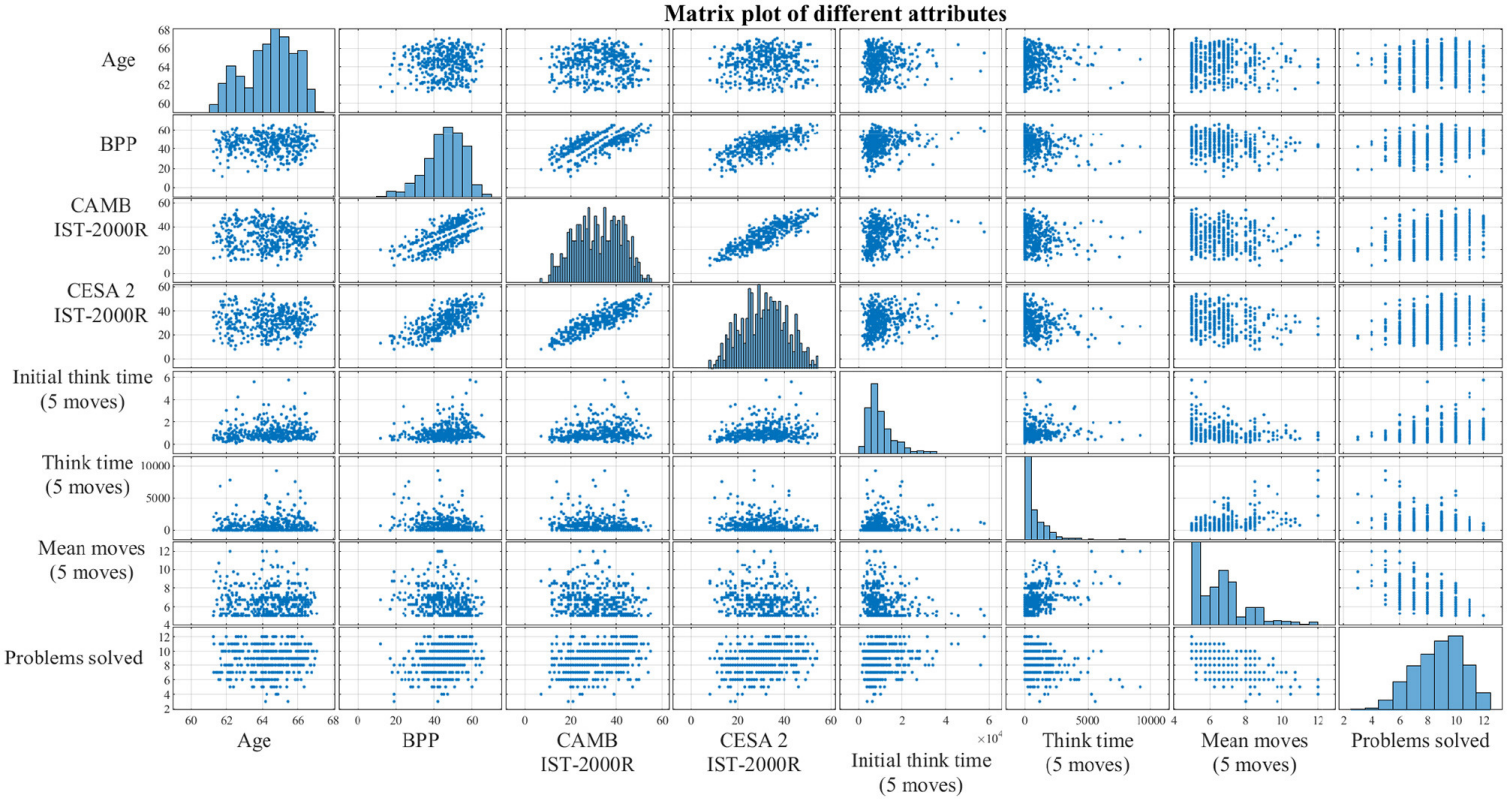
Correlations between the different non-EEG attributes presented as a matrix plot. The diagonal subplots are distributions of each attribute, while the off-diagonal subplots are simply scatter plots between every pair of attributes. The purpose of this matrix plot is to give a clearer understanding of the correlations and the sample distributions. BPP, Børge Priens Prøve; IST-2000R, Intelligens Struktur Test 2000 Revised; CAMB, Copenhagen Aging and Midlife Biobank; Initial think time, SOC mean initial thinking time; Think time, SOC mean subsequent thinking time; Problems solved, SOC problems solved in minimum moves.

A two-sample *t*-test was used to assess the difference between the cognitively stable group (highCog) and the cognitively declining group (lowCog). More specifically, the tested variables include CAMB IST-2000-R (at 56 years) and CESA 2 IST-2000-R (at 60 years) because these variables follow a normal distribution (see also Figure 5). There are 13 SOC variables. However, not all SOC variables are relevant depending on the difficulty of the SOC problem. According to Teubner-Rhodes (2020), the difficulty of a cognitive task is defined by task demand and cognitive ability. If a task is too easy, the subject with a high cognitive ability will not put in sufficient effort. On the other hand, if a task is too difficult, a subject with low cognitive ability will give up more easily (Teubner-Rhodes, 2020). All these factors can lead to inaccurate SOC outcomes, where the EF cannot be assessed.

To select the SOC variables that differentiate the groups the most, a non-parametric statistical test called the Wilcoxon rank-sum test was conducted (Gibbons and Chakraborti, 2014; Ford, 2017). This test was used instead of the two-sample *t*-test because the SOC variables are not normally distributed (see the diagonal elements in Figure 5) and, sometimes, the number of observations is too small. In addition, Bonferroni correction was applied to the two-sample *t*-tests and the Wilcoxon rank-sum tests, respectively. Permutation tests were performed to compare all the topographies of the highCog and lowCog groups, respectively, where the brain areas with a *p*-value lower than 0.05 were highlighted (Wilcox, 2011).

#### 2.4.2 Generalized Linear Model

Generalized Linear Model (GLM) was generated to analyze the relationship between IQ and ASSR and between the EF (SOC variables) and ASSR, respectively. The cognition index (IST-2000R) taken at around 60 years from CESA 2, visit 1, was used as the IQ index. A Z-score transformation was applied to the IQ index, so it had zero mean and a variance of one. From the matrix plot in Figure 5, it is clear to see that IQ above age 18 generally follows a normal distribution, but the number of SOC problems solved in minimum moves is left-skewed, while the mean SOC moves for 4-move problems is right-skewed. After applying the appropriate transformations and link functions, the response variables for IQ (***y***_*IQ*_), SOC mean moves (4 moves) (***y***_*SOCmove*_), and SOC problems solved in minimum moves (***y***_*SOC*_*prob*__) are defined as:


(12) $$\begin{array}{l} y_{IQ}=Z\left(IQ\right)=X\beta_{IQ}, \end{array}$$

(13) $$\begin{array}{l} y_{SOCmove}=\frac{1}{SOC_{move}}=X\beta_{SOCmove}, \end{array}$$

(14) $$\begin{array}{l} y_{SOCprob}=\log\left[\max\left(SOC_{prob}\right)-SOC_{prob}\right]=X\beta_{SOCprob}. \end{array}$$

After back-transforming ***y***_*IQ*_, ***y***_*SOCmove*_, and ***y***_*SOCprob*_, the following equations are used for interpreting the GLM parameters:


(15) $$\begin{array}{l} 𝔼\left[Z\left(IQ\right)\right]=X\beta_{IQ};\text{ (positive and large }\beta \text{ means higher IQ)}, \end{array}$$

(16) $$\begin{array}{l} 𝔼\left(SOC_{move}\right)=\frac{1}{X\beta_{SOCmove}};\\ \text{ (positive and large }\beta \text{ means better performance)}, \end{array}$$

(17) $$\begin{array}{l} 𝔼\left(SOC_{prob}\right)=\max\left(SOC_{prob}\right)-e^{\left\{X\beta_{SOCprob}\right\}};\\ \text{ (positive and large }\beta \text{ means worse performance)}. \end{array}$$

The predictor variables ***X*** and the coefficients **β** are defined as:


(18) $$\begin{array}{l} X\beta =[1+\alpha_{AM}+\alpha_{\text{ITPC}}+\sigma_{AM}+\sigma_{\text{ITPC}}+ \end{array}$$

(19) $$\begin{array}{l} \;\sum_{i=1}^{g=2}group_{i}*(A_{AM}+\mu_{AM}+A_{AM}\mu_{AM}+A_{\text{ITPC}}+\mu_{\text{ITPC}}\\ \;+A_{\text{ITPC}}\;\mu_{\text{ITPC}}+\;P)]\;\beta , \end{array}$$

where ***P*** denote the average ASSR band power values. From fitting the modulation profiles, ***A*** are the amplitudes, **μ** are the latencies, **σ** are the slopes, and **α** are the decays. The variable *group*_*i*_ is categorical, and it represents highCog group as *group*_1_ and lowCog group as *group*_2_. The symbol “*” follows the Wilkinson notation, i.e., a*b = *a* + *b* + *ab*. Please note that only the interactions between ***A*** and **μ** are included directly due to the study's focus.

#### 2.4.3 Cognition classification using Vision Transformer

The Vision Transformer (ViT) base model with 86 million parameters was selected in this study. The model weights were pre-trained on the ImageNet-21k dataset, containing over 14 million images and over 21 thousand classes (Dosovitskiy et al., 2020). The input to the ViT was a 2D-transformed RESS EEG with dimensions 224 × 224. During training, all layers except the final fully connected layer were frozen to prevent overfitting. The 2D-transform, Evoked spectral perturbation (ERSP), is defined as (Mørup et al., 2007):


(20) $$\begin{array}{l} ERSP\left(f,t\right)=\frac{1}{N}\overset{N}{\underset{n}{\sum }}|X\left(f,t,n\right){|}^{2}, \end{array}$$

where *N* is the total number of trials. A 5-fold Cross-Validation (CV) scheme was used to fit the five ViT models on our dataset (see Supplementary Figure S2). Subsequently, the hyperparameters of these five models were optimized using RandomizedSearchCV of the sklearn module combined with the skorch module in Python.

## 3 Results

### 3.1 Statistical tests

The clinical characteristics of the participants are shown in Table 1. There is no significant difference in the BPP test scores at the age of 18. However, there is a significant difference in the later IST tests at age 56 and age 60 and in the subsequent SOC tests. In the group of participants with lower cognitive abilities, there were significantly more movements made to complete the task (*p* = 0.001), and they solved fewer problems within the allotted time *p* = 0.000168). The correlations between some of the non-EEG attributes are shown using scatter plots and histograms in Figure 5. It was based on this initial data visualization that the statistical test types were decided.

**Table 1:** Difference between the cognitively stable group (highCog) and the cognitively declining group (lowCog) expressed in P-values.

**Participants (*n* = 178)**	**Group 1 (highCog)**	**Group 2 (lowCog)**	**Difference (*p*-value)**
No. of participants	83	95	-
Mean BPP (18 yrs)	46.4	45.4	0.182^‡^
Mean IST (56 yrs)	40.9	23.9	8.80·10^−40^^†^^*^
Mean IST (60 yrs)	37.6	25.5	7.70·10^−18^^†^^*^
SOC moves for 4-move problems (mean)	4.94	5.40	0.00170^‡^^*^
SOC problems solved in min moves (mean)	9.45	8.31	0.000168^‡^^*^

For normally distributed variables, including age and the two IST-2000R scores, a two-sample *t*-test was applied to calculate the *P*-value. A Wilcoxon rank-sum test was then done for the remaining variables, which follow a skewed distribution.

† Calculated using two-sample *t*-test.

‡ Calculated using Wilcoxon rank-sum test.

* Significant (*P*-value < 0.0031).

### 3.2 Complex demodulation

**Figure 6:**
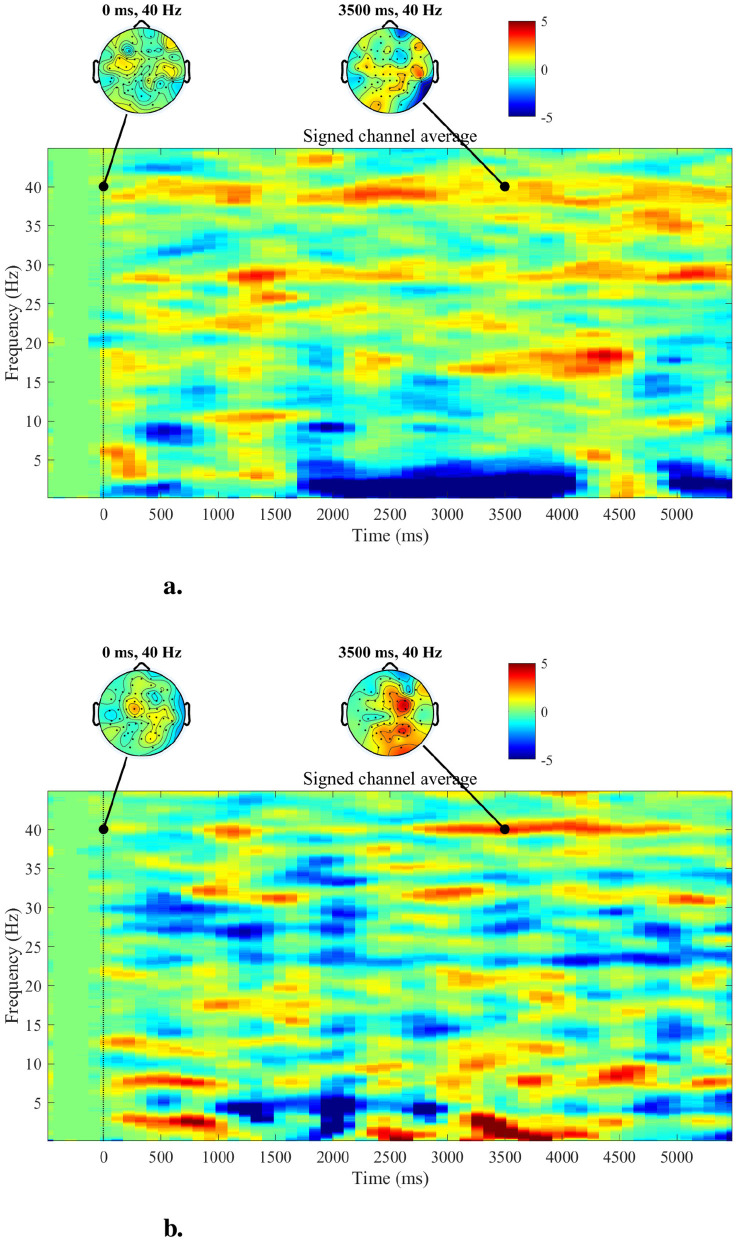
Topographies and spectrograms for a highCog subject **(A)** and a lowCog subject **(B)**, respectively. The topographies indicate that the highCog subject has visibly weaker but more stable responses in the overall brain, while the lowCog subject has particularly strong responses in the central parts. Furthermore, the spectrograms indicate that the highCog subject has a stronger ability to maintain the 40 Hz ASSR than the lowCog subject, who has a more diffuse power distribution.

**Figure 7:**
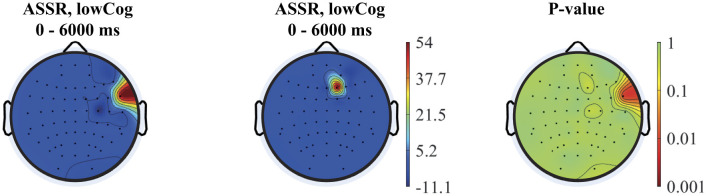
From left to right: averaged topographies computed for all highCog and lowCog subjects along with the *p*-values from the permutation tests. The figure depicts statistical differences mostly around the temporal cortex, while other parts of the cortex showed similar activation patterns comparing the two groups.

The topographies of the 40 Hz ASSR power and spectrogram in the time domain are depicted for two subjects of the lowCog and highCog group separately for qualitative assessment (see one example in Figure 6). We found a consistent qualitative difference between the two groups. The 40 Hz power of highCog subject in Figure 6A was more focused at the temporal region, whereas the lowCog subject in Figure 6B has a stronger and more diffuse 40 Hz power involving a larger area on the cortex. Additionally, this difference is reflected by the averaged topographies presented in Figure 7. Here, we found a strong and highly significant frontotemporal response for the highCog group, which is completely absent for the lowCog group. On the other hand, the lowCog group has shown a strong frontocentral response. The *p*-values in Figure 7 were obtained from exploratory permutation tests between both groups. A similar statistical comparison can also be made for the spectrograms, where each pixel from the averaged highCog spectrogram is compared to the corresponding pixel from the averaged lowCog spectrogram. However, this part is omitted because of redundancy and heavy computation.

**Figure 8:**
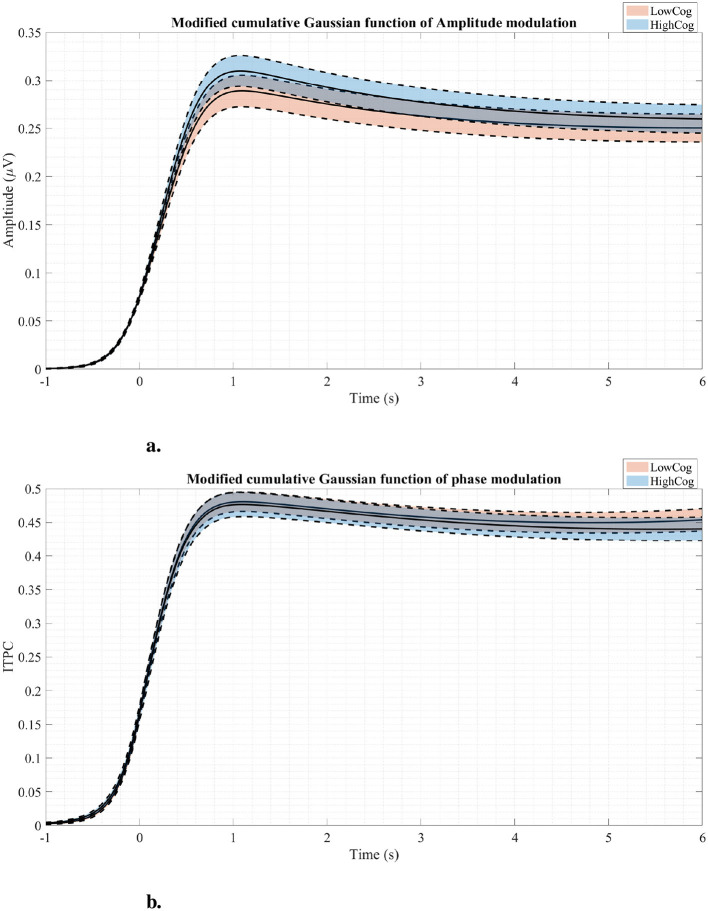
Amplitude modulation profiles **(A)** and phase modulation profiles **(B)** fitted using the MCGF for both cognition groups. The solid lines represent the average modulation curves, while the dashed lines represent standard errors. Generally, the highCog group seems to have a larger amplitude modulation than the lowCog group.

The MCGF was fitted to the mixed region RESS EEG and can be seen in Figure 8. The AM MCGF profiles in Figure 8A show that the highCog subjects have larger amplitudes than the lowCog subjects. On the other hand, the ITPC MCGF profiles in Figure 8B showed a delayed latency modulation for lowCog individuals. The raw average AM and ITPC profiles are shown in Supplementary Figure S1).

### 3.3 Generalized linear models

Two GLMs are fitted to the mixed region, while one GLM is fitted to the frontal region. The response variables are ***y***_*IQ*_, ***y***_*SOCmove*_, and ***y***_*SOC*_*prob*__, respectively. The *R*^2^ value and the log-likelihood indicate how good the model is. The closer the *R*^2^ value is to 1, the more variability is explained by the model (Montgomery, 2017). The more positive the log-likelihood is, the better fitted the model is Madsen (2007). For the mixed region, the GLMs of ***y***_*IQ*_ and ***y***_*SOCmove*_ are greatly significant compared to the constant models (for ***y***_*IQ*_: *F* = 23.5, *p* = 3.56 × 10^−20^, *R*^2^ value = 0.452, and loglikelihood = −199; for ***y***_*SOCmove*_: *F* = 4.75 and *p* = 0.000430, *R*^2^ value = 0.126, and loglikelihood = −231). In the GLM for ***y***_*IQ*_, the AM magnitude (*Estimate* = 5.24 ± 1.33, *t* = 3.93, *p* = 0.000123) is proportional to IQ. On the other hand, the magnitude of ITPC is also proportional to IQ (*Estimate* = 1.99 ± 0.738, *t* = 2.69, *p* = 0.00779). Lastly, the average ASSR band power (*Estimate* = −16.5 ± 4.45, *t* = −3.70, *p* = 0.000290) is negatively proportional to IQ, indicating that the larger the ASSR power is, the worse the cognition is (see Table 2).

**Table 2:** IQ GLM for the mixed region.

**Response variable**	**Predictor variable**	**Parameter estimate**	**Standard**	***t*-statistic**	**Pr(>|*t*|)**
***y***_*IQ*_	Intercept_*model*_	–2.03	0.365	–5.57	9.84·10^−8^
	Intercept_*highCog*_	2.94	0.423	6.96	7.36·10^−11^
	Magnitude (ITPC)	–1.41	0.506	–2.79	0.00588
	Slope (AM)	1.35	0.465	2.91	0.00408
	Magnitude (AM) · lowCog	5.24	1.33	3.93	0.000123
	Magnitude (ITPC) · lowCog	1.99	0.738	2.69	0.00779
	ASSR power · lowCog	–16.5	4.45	–3.70	0.000290

The model is given in Equation 15. *R*^2^ value = 0.452, and loglikelihood = −199.

In the GLM for ***y***_*SOCmove*_, the magnitude of AM is once again positive and significant (*Estimate* = 0.124 ± 0.0409, *t* = 3.04, *p* = 0.00276) (see also Equation 16). The more steps the participant needs to complete the test, the lower the AM magnitude.

Regarding the number of SOC problems solved in minimum moves ***y***_*SOC*_*prob*__, the GLM from the frontal region is the most informative. This model is significant compared to the constant model (χ^2^ = 26.3, *p* = 2.69 × 10^−5^, *R*^2^ value = 0.128, and loglikelihood = −339). Moreover, the latency coefficent of AM is negative and significant for both groups (*Estimate* = −2.41 ± 0.671, *t* = −3.59, *p* = 0.000325). According to Equation 17, a low AM latency means that the term *e*^***Xβ***^ becomes smaller, leading to more SOC problems solved (better EF). This model is shown in Table 3.

**Table 3:** *SOC*_*prob*_ GLM for the frontal region.

**Response variable**	**Predictor variable**	**Parameter estimate**	**Standard error**	**t-statistic**	**Pr(>|*t*|)**
***y***_*SOCprob*_	Intercept_*model*_	1.35	0.0641	21.0	7.18·10^−98^
	Latency (AM)	–2.41	0.671	–3.59	0.000325
	Magnitude (AM) · highCog	–2.11	0.584	–3.61	0.000302
	Magnitude (AM) · Latency (AM) · highCog	10.7	2.52	4.23	2.38·10^−5^
	Latency (AM) · lowCog	2.18	0.662	3.30	0.000969

The model is given in Equation 17. *R*^2^ value = 0.128, and loglikelihood = −339.

Comparing the GLM results to Figure 8A, it is clear that the highCog subjects exhibit a larger AM amplitude than the lowCog subjects. This aligns well with Tables 2, 4. On the other hand, Figure 6 also aligns well with Table 2, where a strong power distribution is present for the lowCog subject, worsening his cognition.

**Table 4:** *SOC*_*move*_ GLM for the mixed region.

**Response variable**	**Predictor variable**	**Parameter estimate**	**Standard error**	***t*-statistic**	**Pr(>|*t*|)**
***y***_*SOCmove*_	Intercept_*model*_	0.172	0.00790	21.8	3.86·10^−51^
	Intercept_*highCog*_	0.0572	0.0149	3.85	0.000164
	ASSR power	-0.444	0.127	-3.50	0.000598
	Latency (AM) · highCog	-0.107	0.0502	-2.13	0.0344
	Magnitide (AM) · Latency (AM) · highCog	0.284	0.121	2.34	0.0205
	Magnitude (AM) · lowCog	0.124	0.0409	3.04	0.00276

The model is given in Equation 16. *R*^2^ value = 0.126, and loglikelihood = −231.

### 3.4 Cognition classification using Vision Transformer

The performance of the trained and optimized ViT models is shown in Table 5. This result shows that each fold generally yields a performance of around 50%.

**Table 5:** ViT models trained and tested on the mixed region using a five-fold CV setup.

**ERSP on mixed region**	**Fold 1**	**Fold 2**	**Fold 3**	**Fold 4**	**Fold 5**	**Average**
Accuracy (%)	50.0	55.9	52.9	47.1	52.9	51.8
Specificity (%)	37.5	68.8	31.3	50.0	56.3	48.78
Sensitivity (%)	61.1	44.4	72.2	44.4	50.0	54.42

Hyperparameters are fine-tuned for all models.

## 4 Discussion

The main finding of the study is that men with declining cognitive function show prolonged phase modulation, higher amplitude, and a larger area of entrainment in their ASSR. These distinct declines are linked to significantly poorer EF, as measured by the SOC task. Other recent studies have shown that increased 40 Hz ASSR power correlated to worse cognitive performance in patients with Alzheimer's Disease (AD) compared to Mild Cognitive Impairment (MCI) and controls (Tada et al., 2020; Van Deursen et al., 2011). In our study, we used healthy subjects without clinical symptoms of MCI or dementia. Nevertheless, we still found significant increased ASSR power in our low cognition group. This finding suggests that the decline observed from young to middle age possibly represents preclinical cognitive decline.

### 4.1 EF and 40 Hz ASSR

Previous studies on healthy young individuals have highlighted a positive correlation between EF and strength and synchronicity measures of 40 Hz ASSR (Parciauskaite et al., 2019). The ASSR at 40 Hz might represent top-down mechanisms that are related to cognitive functioning (Parciauskaite et al., 2019; Müller et al., 2009). Alterations in gamma-range ASSR indicate the degree of attentional control and the capacity to temporarily store and manipulate information. These abilities are essential for a broad spectrum of complex cognitive activities, in both healthy individuals and those with impairments (Parciauskaite et al., 2021). ASSRs are considered to represent purely sensory processes and to reflect the integrity of auditory circuits. Additionally, they are thought to index globally synchronized neural activity and facilitate information transfer (Tada et al., 2016; Teale et al., 2003). Moreover, 40 Hz ASSRs are perceived as an index of neurochemical excitation/inhibition balance in the brain maintained by N-methyl-d-aspartate (NMDA) and γ-aminobutyric acid (GABA) systems, as shown in animal studies (Vohs et al., 2010; Sivarao et al., 2016; Sullivan et al., 2015). Changes in the NMDA/GABA balance in the prefrontal cortex causes delay in a persons ability to respond (Auger and Floresco, 2017).

### 4.2 Delayed synchronization

However, our study is the first describing delayed neural assembly during auditory synchronization of healthy aging individuals and connecting the delayed phase synchronization to advanced cognitive aging. This association potentially indicates that the late-latency gamma in response to auditory 40 Hz stimulation might index abilities for planning and problem-solving. This finding correlates to our former studies on visually evoked steady-state responses where we confirmed age-related changes in gamma oscillations, including a posterior-to-anterior shift in oscillatory activity and a reduction in gamma band synchrony (Bakhtiari et al., 2023). These alterations in gamma power precede potential changes in alpha band power. Furthermore, our data underscore the critical role of gamma synchrony in maintaining cognitive functions (Bakhtiari et al., 2023). The latency of gamma frequency Steady-State Visually Evoked Potentials (SSVEPs) also increases with cognitive decline. This indicates that the disruption of SSVEP facilitation initially occurs at gamma frequencies, followed by alpha frequencies (Richard et al., 2020). We hypothesize that our findings on delayed or unstable phase synchronization of ASSR in cognitive and age-related cognitive decline may result from a reduced ability to maintain and coordinate perceptual information. This is consistent with previous meta-analyses on inhibition deficits in older adults (Rey-Mermet and Gade, 2018; Hsieh et al., 2012).

### 4.3 Cognitive decline

Our study population is all healthy individuals without any clinical signs of cognitive decline. However, similarities of our present neurophysiological, earlier imaging findings (Rosemann and Thiel, 2020) highlight distinct changes in our declining group that are quite similar to and eventually could precede MCI and AD. Cognitive decline is a result of multiple life factors as previous studies on the same subjects also indicated that; decreases in IQ, less physical activity, and poorer mental health were associated with decreased whole brain volumes (Zarnani et al., 2020). Our study population differs based on the relative decline between 18 to 56 years of age, which was in line with continuing or discontinued education in our two groups. Recent large-scale community-based, longitudinal clinical, and pathological studies demonstrated that early-life cognitive enrichment was associated with lower AD pathology indices and slower late-life cognitive decline (Oveisgharan et al., 2020).

Recently, a cognitive reserve hypothesis has been proposed to explain how individuals with similar neuropathological conditions differ substantially in their ability to make efficient use of brain reserve during tasks (Stern et al., 2019). Intelligence (Alexander et al., 1997) and higher education (Amieva et al., 2014), occupational level (Staff et al., 2004), participation in leisure activities (Scarmeas et al., 2001), and social networking (Fratiglioni et al., 2000) are considered to be contributing factors to the cognitive reserve. In our study, subjects with declining intelligence between 18 and 56 years did worse on cognitive tests in late life, strengthening the hypothesis of protective factor of brain reserve against cognitive decline.

### 4.4 Auditory processing

Our finding on relatively delayed network synchronization during auditory stimulation and worsening EF of low-performing individuals are in line with clinical findings of hearing impaired, who require longer latencies to make accurate perceptual judgments (Tun et al., 2010). The connection between hearing impairment, auditory processing and cognition is evident, and rather complex. Aging results in pathological and physiological changes in both peripheral and central auditory systems. Approximately, 83% of adults 70 years and above suffer from peripheral hearing loss (Cruickshanks et al., 1998). Peripheral hearing loss not only affects the auditory processing of speech sounds but also the higher-level cognitive functions required to process linguistically demanding stimuli (Jayakody et al., 2018; Powell et al., 2021). Hearing thresholds obtained from pure tone audiometry and ASSR were found to be significantly correlated in a cohort consisting of participants with normal hearing or mild hearing loss (Tarawneh et al., 2022). The activation patterns, summarized in the averaged topographies (Figure 7), revealed significant differences in activation, particularly in the temporal lobe, between the high and low cognition groups.

### 4.5 GLM and ML model

From a modeling viewpoint, the *R*^2^-values of the GLMs range only between 0.13 and 0.45, which hardly indicates a good fit. However, it is still a slight improvement compared to another study, which only achieved a *R*^2^-value of 0.12 while using a similar approach (Richard et al., 2020). Our second hypothesis, which is that we can predict low-performing individuals with deep learning at preclinical cognitive decline, showed low accuracy in our healthy aging cohort. Nonetheless, we did optimize the ML model to its limit while taking great care to avoid information leakage (train-test-overlap) and overfitting. At the current time, no other study has demonstrated a more accurate prediction of a clinically healthy aging cohort. Nevertheless, this result emphasizes the difficulty in assessing and thus preventing MCI and AD at an early stage using ASSR.

### 4.6 Strength and limitation of the study

The strength of this study lies in its prospective longitudinal design, following participants from birth to the age of 68, which provides valuable insights into cognitive function over a significant portion of the lifespan. A notable advantage is the focus on individuals with declining cognitive function but without clinical signs of dementia, a group that is less frequently studied. Additionally, the consistency of data collection, with the same technicians using identical equipment and parameters to record ASSR, enhances the reliability of the findings. However, the study has limitations. For example, no female participants are included in this study because a mandatory military service draft is not required of them. Moreover, there is a potential selection bias due to the inclusion process, as participation depended on telephone contact with individuals previously enrolled in the cohort. This approach may have disproportionately attracted individuals with stable life circumstances, potentially excluding those experiencing depression or challenging life events.

ASSR at 40 Hz is also a small signal with possible intra- and inter-individual variability, making it challenging to compare individuals with only slight differences in cognitive performance. A larger dataset would improve the robustness of such comparisons. Furthermore, the relatively small sample size limits the application of advanced methods such as deep learning or ML algorithms. We will continue to collect data to assess correlations between EF and ASSR in late life in the future. As for the cognitive classifier, we will experiment with other ML models and, if necessary, appropriate data augmentation methods to improve the classification result.

## 5 Conclusion

In this study, we analyzed a longitudinal database of healthy male Danish volunteers. By fitting Generalized Linear Models (GLMs) to a mixed brain region consisting of temporal, central, and parietal electrodes, we identified strong correlations between neural assembly (AM magnitude), synchronization consistency (ITPC magnitude), and average ASSR power with EF. More specifically, smaller neural assemblies, higher ASSR power, and larger areas of entrainment were highly correlated to low cognitive outcomes. Additionally, a GLM from the frontal region revealed a strong correlation between response latency (AM latency) and EF, indicating that longer AM latency is predictive of poorer EF. Finally, an ensemble of five Vision Transformer (ViT) models demonstrated low accuracy in predicting cognitive decline, underscoring the challenges in developing an effective cognitive classifier and the necessity for ongoing research.

## Data Availability

The datasets presented in this article are not readily available because the data in the article is considered confidential because it involves sensitive patient information, which, despite the patients' consent for use within the center, requires additional safeguards for broader use. Protecting patient privacy is a key ethical responsibility, and further dissemination or secondary use of the data must be reviewed by an ethical committee to ensure compliance with legal and ethical standards. This oversight ensures that patient consent, privacy, and confidentiality are upheld, safeguarding against any misuse or unauthorized access to personal health information. Requests to access the datasets should be directed to krben@regionsjaelland.dk.
