# Supplementary material for: Auditory steady state response can predict declining EF in healthy elderly individuals
Source: Front Aging Neurosci. 2025 Feb 4;17:1516932. doi: 10.3389/fnagi.2025.1516932 (PMC11832718; doi:10.3389/fnagi.2025.1516932)
Supplement: Supplementary file 1 [file Data_Sheet_1.pdf]

Supplementary Material

1 SUPPLEMENTARY TABLES AND FIGURES

1.1 Figures

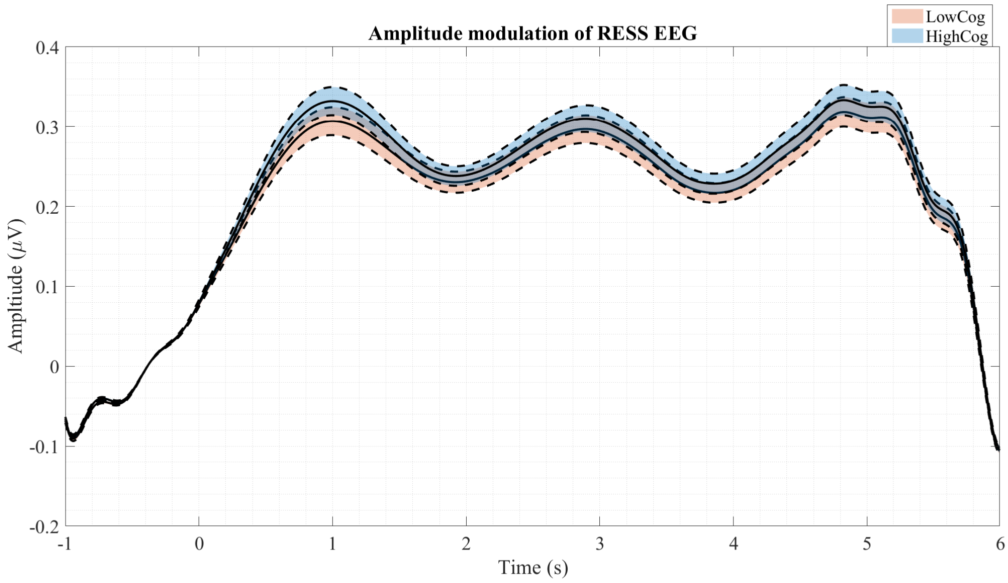

Figure S1a

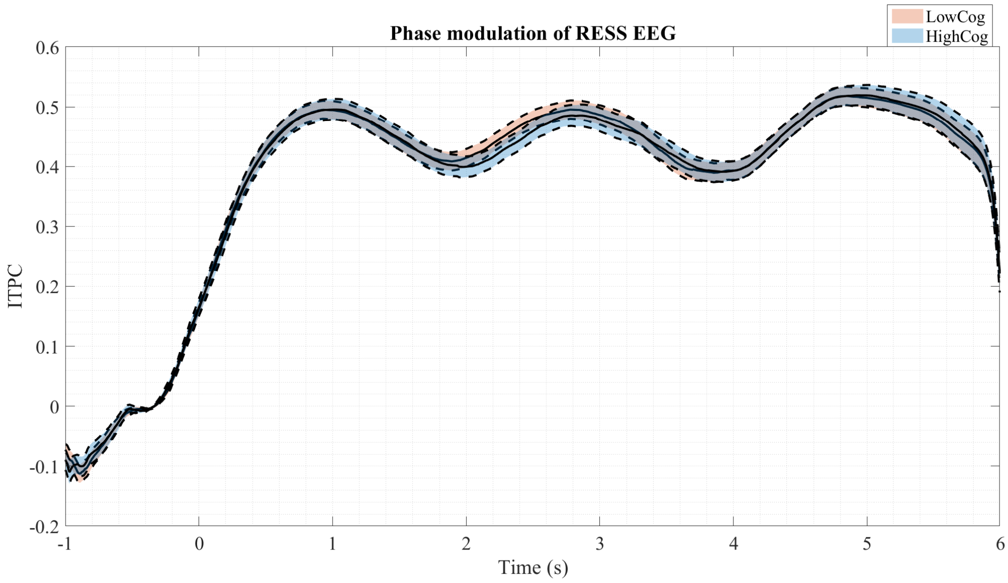

Figure S1b

Figure S1: The amplitude modulation profiles **(a)** and phase modulation profiles **(b)** for both cognition groups.

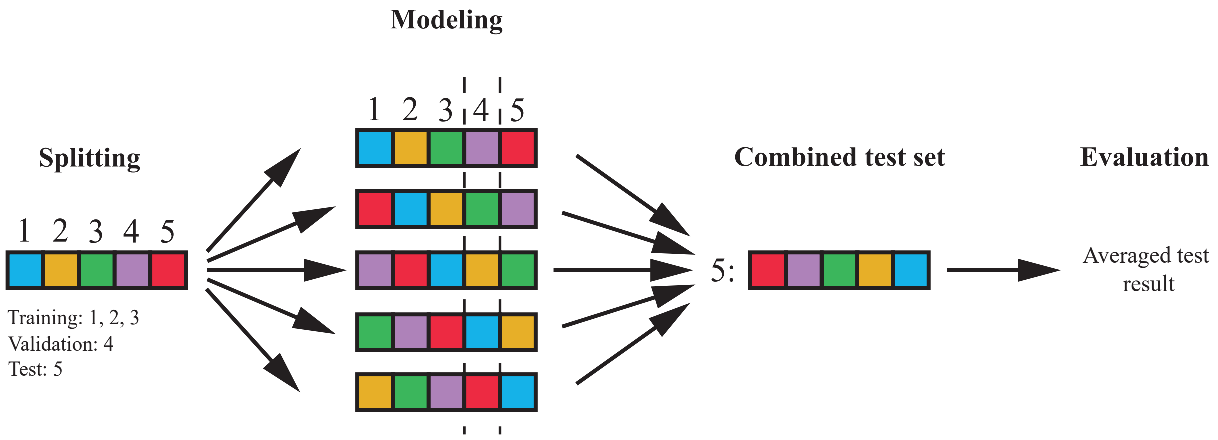

Figure S2: The five-fold Cross-Validation scheme used for training the Vision Transformes.
